# The Road to Malignant Cell Transformation after Particulate Matter Exposure: From Oxidative Stress to Genotoxicity

**DOI:** 10.3390/ijms24021782

**Published:** 2023-01-16

**Authors:** Miguel Santibáñez-Andrade, Ericka Marel Quezada-Maldonado, Andrea Rivera-Pineda, Yolanda I. Chirino, Claudia M. García-Cuellar, Yesennia Sánchez-Pérez

**Affiliations:** 1 Subdirección de Investigación Básica, Instituto Nacional de Cancerología, San Fernando No. 22, Tlalpan, México City CP 14080, Mexico; 2 Departamento de Biología Celular, Centro de Investigación y de Estudios Avanzados del IPN (CINVESTAV), Av. IPN No. 2508 Col. San Pedro Zacatenco, México City CP 07360, Mexico; 3 Unidad de Biomedicina, Facultad de Estudios Superiores Iztacala, Universidad Nacional Autónoma de México, Los Reyes Iztacala, Tlalnepantla CP 54090, Mexico

**Keywords:** oxidative stress, genotoxicity, cancer, air pollution, particulate matter

## Abstract

In cells, oxidative stress is an imbalance between the production/accumulation of oxidants and the ability of the antioxidant system to detoxify these reactive products. Reactive oxygen species (ROS), cause multiple cellular damages through their interaction with biomolecules such as lipids, proteins, and DNA. Genotoxic damage caused by oxidative stress has become relevant since it can lead to mutation and play a central role in malignant transformation. The evidence describes chronic oxidative stress as an important factor implicated in all stages of the multistep carcinogenic process: initiation, promotion, and progression. In recent years, ambient air pollution by particulate matter (PM) has been cataloged as a cancer risk factor, increasing the incidence of different types of tumors. Epidemiological and toxicological evidence shows how PM-induced oxidative stress could mediate multiple events oriented to carcinogenesis, such as proliferative signaling, evasion of growth suppressors, resistance to cell death, induction of angiogenesis, and activation of invasion/metastasis pathways. In this review, we summarize the findings regarding the involvement of oxidative and genotoxic mechanisms generated by PM in malignant cell transformation. We also discuss the importance of new approaches oriented to studying the development of tumors associated with PM with more accuracy, pursuing the goal of weighing the impact of oxidative stress and genotoxicity as one of the main mechanisms associated with its carcinogenic potential.

## 1. Introduction

During the past decades, air pollution has become a global and local problem, negatively impacting human health. These effects have been widely studied in high-population-density cities, where the increase in pollutant emissions is associated with daily activities, such as transportation and industrial activities. Epidemiological studies have reported strong associations between air pollution and the arise of chronic-degenerative diseases. From these studies, it has been demonstrated that particulate matter (PM) released into the environment by vehicles and industries increases the incidence of multiple respiratory diseases, such as asthma, chronic obstructive pulmonary disease, and, in the long term, cancer [1,2,3,4]. On the other hand, toxicological studies have described the biological mechanisms altered by PM in numerous models and how these cellular alterations contribute to uncontrolled cell proliferation and, thus, carcinogenesis [5,6,7,8]. One aspect that becomes relevant is the role of oxidative stress caused by PM in carcinogenesis, because an immediate effect of PM exposure in the human body and, therefore, in cells, is the generation of reactive oxygen species (ROS). Oxidative stress plays a determinant role in cancer, but how important is during the different stages of malignant transformation still represents a challenge in biomedical research.

In this review, we summarized the evidence regarding the effect of PM-mediated oxidative stress during malignant cell transformation, emphasizing the carcinogenic effects of ROS, once there is little evidence regarding PM and lipoperoxidation-protein oxidation oriented to cancer. Our approach is oriented from a perspective widely accepted in the study of the biological mechanisms associated with carcinogenesis: the “multistep carcinogenesis mechanisms” perspective proposed by J. Carl Barret [9,10,11]. Finally, we will discuss the importance of novel approaches under the context of PM risk assessment, capable of elucidating the carcinogenic potential of PM-mediated oxidative stress.

### 1.1. Reactive Oxygen Species and Oxidative Stress: A Source of Cellular Damage

The term “oxidative stress” refers to a physiological state characterized by an imbalance between intracellular free radicals and oxidants and the cellular antioxidant defense in favor of the first ones, causing damage to different cellular components and changing the functioning of key biomolecules [12,13]. Oxidative stress emerges from a disturbance or decrease in the activity of endogenous protective systems that neutralize and eliminate oxidative attack, as well as from the increase in the production rate of radical species, which leads to a saturation of the defense mechanisms.

Free radicals are molecules that have an extremely short half-life (on the order of a few seconds) because they present an unpaired electron in the external orbit which quickly reacts with other molecules causing oxidative damage [14]. All compounds that can accept electrons are oxidizing agents, in contrast, a reducing agent donates electrons, the process between one reaction and another is called a redox reaction and in biological terms, these concepts are determined as pro-oxidant and antioxidant elements, respectively [15]. Derivatives of the radicals are oxy radicals such as reactive oxygen species (ROS) and reactive nitrogen species (RNS). ROS include all metabolites of molecular oxygen (O_2_) that are reduced to different intermediate species such as superoxide anion (O_2_•-), hydroxyl radical (OH•) the most reactive ROS, as well as a to non-radical molecules such as hydrogen peroxide (H_2_O_2_) which, although it is not a free radical, is highly oxidant and causes damage to the cell at a relatively low concentration [16,17].

Under basal conditions, free radicals are formed in aerobic processes that use oxygen. For that reason mitochondria, peroxisomes, and the endoplasmic reticulum produces ROS in various metabolic activities [18]. During ATP production, in the electron transport chain, the reduction of molecular oxygen (O_2_) to water produces ROS as follows:

O_2_ + e ^−−^ + H^+^ → HO_2_^•^

HO_2_ • → H^+^ + O_2_^•−^

O_2_^•−^ + 2H^+^ + e^−−^ → H_2_O_2_

H_2_O_2_ + e^−−^ → OH^−−^ + OH^•^

OH^•^ + H^+^ + e ^−−^ → H_2_O

The superoxide radical (O_2_^•−^) is produced in the mitochondria in different complexes including complex I, complex III, and glycerol 3-phosphate dehydrogenase. Manganese superoxide dismutase (Mn-SOD) as well as Cu- and Zn-SOD converts the O_2_^•−^ to hydrogen peroxide (H_2_O_2_), which can further be converted by mitochondrial aconitase to a hydroxyl radical (OH^•^) by Fenton-types reactions, in which H_2_O_2_ is dismutated by the oxidation of metal ions principally Fe_2_^+^ or Cu^+^ (Fe^2+^ + H_2_O_2_ → Fe^3+^ + OH^−^ + OH•). Finally, this radical can be converted to water (H_2_O) for elimination. OH^•^ is the most reactive and damaging ROS molecule for cells, due to its high reactivity. The OH• interacts in the place of its production with the molecules that surround it closely [12,19,20].

One more site of ROS production in mitochondria is the microsomal cytochrome P450 enzyme complex which metabolizes different organic substrates producing H_2_O_2_ [21]. In addition, several other mammalian proteins generate ROS such as xanthine oxidase, a phase I enzyme that induces oxidation, reduction, and hydrolysis of different compounds producing O_2_^•−^, OH^•^ and H_2_O_2_ [22], as well as succinate dehydrogenase and monoamine oxidase that produce O_2_^•−^ and H_2_O_2_ respectively [19,23,24]. ROS can be produced by endogenous oxidants including nicotinamide adenine dinucleotide phosphate (NADPH), angiotensin II and myeloperoxidase responsible for the generation of O_2_^•−^ [25,26]. However, the most common source of oxidants are exogenous agents derived from different environmental/lifestyle factors to which the human body is commonly exposed, such as UV radiation which produces O_2_^•−^ and H_2_O_2_, in addition to tobacco smoke and environmental pollutants that in general produces both O_2_^•−^, H_2_O_2_ and OH^•^ [27,28,29,30]. On the other hand, peroxisomal beta-oxidation and activated phagocytic cells also produce ROS [31].

Unlike ROS generators, antioxidants are molecules that are capable of delaying or preventing the oxidation of different substrates, maintaining the redox balance, since they prevent the formation of ROS or eliminate them [27]. The principal endogenous antioxidant defense enzymes include superoxide dismutase (SOD) which removes O_2_^•−^, catalase (CAT), glutathione peroxidase (GPx), and glutathione reductase (GT) which catalyzes detoxification of H_2_O_2_ preventing OH^•^ generation, these molecules are identified as a “first line of defense” and act in the presence of cofactors such as copper, zinc, manganese, and iron. While nonenzymatic compounds are the “second line of defense” and work by disrupting free radical chain reactions. They are represented by endogenous metabolic compounds such as glutathione and antioxidant enzyme cofactors (coenzyme Q10). On the other hand, exogenous non-enzymatic antioxidants include vitamin C (ascorbic acid), vitamin E (α-tocopherol) carotenoids, and bioflavonoids [25,26,32,33,34,35,36].

### 1.2. Biomolecules Impairment and Cellular Consequences of Oxidative Stress

ROS in low amounts are products of basal cellular metabolism and modulate the function of all classes of biomolecules. ROS are required as signaling molecules for different subcellular processes and signaling pathways, such as enzyme activation, disulfide bond formation during the folding of new proteins in the endoplasmic reticulum, signal transduction, and gene expression through triggering transcription factors. Moreover, its generation is essential in the defense mechanism of phagocytes [37,38]. However, because ROS targets almost all cell substrates, in high concentrations, these radicals can interact with most organic molecules and modulate their function, producing important alterations in many cellular macromolecules, such as nucleic acids, proteins, amino acids, and lipids, which lead to the appearance of more reactive products, which are recognized as biomarkers of oxidative stress [33].

**Figure 1:**
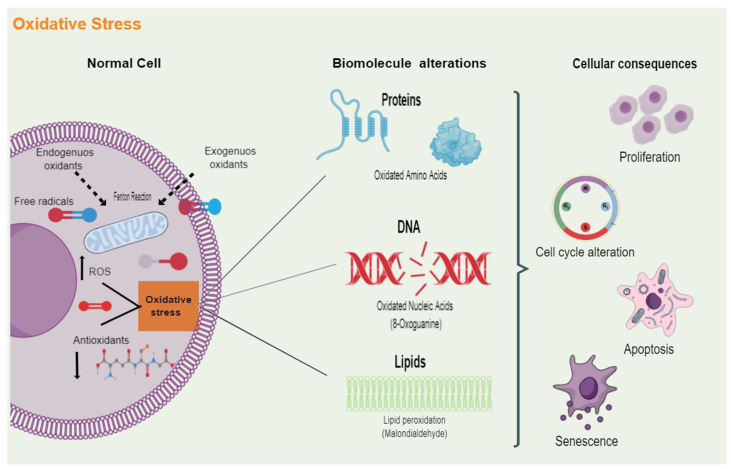
Cellular damage induced by oxidative stress. Multiple endogenous and exogenous sources produce free radicals, which are molecules that contain one or more unpaired electrons. When an imbalance arises between the oxidative molecules present in cells, such as reactive oxygen species (ROS), and the antioxidant defenses that neutralize and eliminate these reactive radicals, oxidative stress is generated. Excess oxidative stress can play a dominant role in protein, cell membrane phospholipids, and DNA damage. The attack of ROS on these biomolecules alters their functions and, ultimately, will lead to the dysregulation of different cellular processes, including proliferation, cell cycle, cell death, and senescence.

In lipids, ROS generate reactive aldehydes such as malondialdehyde (MDA) and 4-hydroxynonenal. About proteins, ROS causes alterations in their conformation due to backbone fragmentation, side-chain oxidation, and the unfolding or misfolding of their structure. In addition, ROS-induced effects on the nucleic acid, ranging from DNA-protein crosslinking, alterations in purine and pyridine base structure, and single and double-strand breaks. The biomolecule alterations induced by oxidative stress can result in cell death or on the contrary in the induction of proliferation, cell survival, alterations of the cell cycle, or senescence (Figure 1). Moreover, oxidative stress predisposes to the generation of mutations due to the damage they cause to DNA for which ROS are considered inducers of malignant transformation of cells [39,40]. The impact of oxidative stress inside the body depends on multiple factors: the type of oxidant, the intensity of its production, the part on the cellular compartment in which the ROS are generated, the capacity of the repair systems, and, most important, the length of redox imbalances [17,41]. According to this, ROS have been implicated in several chronic diseases such as atherosclerosis, immune system dysfunctions, neurodegenerative pathologies (e.g., Alzheimer’s and Parkinson’s), diabetes mellitus, and, importantly, cancer [42,43,44,45]. Oxidative stress has been identified in the early phases of these diseases, which suggests that their etiology is associated with different types of damage [17,31].

## 2. Role of Oxidative Stress in Malignant Cell Transformation

Elevated levels of ROS promote a variety of biological effects required for the development, establishment, and progression of tumors associated with DNA, proteins, and lipids damage [46,47]. It has been described that cancer cells are characterized by higher amounts of ROS compared to normal cells [48,49]. The carcinogenic effect of oxidative stress is broadly related to its effect on DNA. ROS structurally modify both purine and pyrimidine bases, inducing multiple lesions such as DNA abasic sites, DNA base modifications (8-oxoadenine, 2-hydroxy adenine cytosine glycol, 5-hydroxycytosine and 8-hydroxy-2′-deoxyguanosine (8-OHdG), single-strand DNA breaks, and double-strand DNA breaks [50,51,52]. From the types of DNA alterations induced by oxidative stress, 8-OHdG is, by far, the most relevant and predominant lesion. 8-OHdG has been widely used as an oxidative stress biomarker and has been reported to be increased in various types of cancer [53,54].

Related to these, DNA damage repair pathways have been considered as other types of defense of the cells because they remove the damage that has already been generated by ROS, acting as an alternative mechanism to avoid toxic consequences [55], however, when DNA damage caused by ROS is not repaired correctly, the consequence is the presence of replication errors across DNA double-strand, predisposing cells to the generation of genomic instability and DNA alterations such as point mutations, insertions, deletions, and chromosomal translocations, features required for cell transformation because these alterations could cause oncogene activation and tumor suppressor gene inactivation [55,56,57,58]. Furthermore, oxidative stress can modulate gene expression (directly or indirectly), alter epigenetic mechanisms (by inducing changes in methylation or non-coding RNA molecules), and even modify posttranslational markers (for example deregulated protein phosphatases) [59,60,61,62]. These altered events affect multiple signaling pathways, comprising cellular homeostasis, and predisposing cells to malignant transformation.

**Figure 2:**
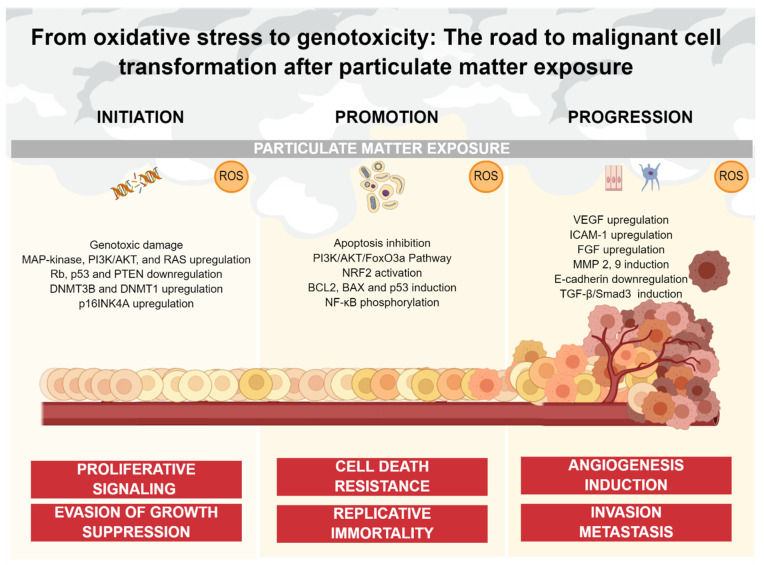
PM-mediated oxidative stress and its impact on cancer continuum. Oxidative stress caused by PM exposure is involved in multiple stages of the carcinogenic process. ROS can induce genotoxic damage, as well as non-genotoxic damage such as upregulation of oncogenic pathways and downregulation of tumor-suppressor components oriented to sustain proliferation and block anti-growth signals (initiation). ROS also inhibits apoptotic signaling through multiple pathways, promoting cell death resistance and replicative immortality (promotion). Finally, ROS upregulate multiple factors and pathways oriented to the acquisition of an invasive phenotype, as well as the generation of a blood vessel network from a pre-existing one (progression).

Therefore, increased levels of ROS can modulate many pathways with carcinogenic potential, resulting in the activation of pro-survival signaling, a decrease of tumor suppressor gene function, elevated glucose metabolism, and resistance to hypoxia. These pathways conduce to cellular proliferation, apoptosis evasion, angiogenesis, as well as invasion and metastasis, leading to the initiation, promotion, and progression of neoplasms [47,56,63]. For these reasons, the main axis of this review is to summarize and discuss the evidence that supports the concept of oxidative stress as an important driving force in cancer development associated with PM exposure. To accomplish our aim, we discuss the scientific reports that demonstrate the role of oxidative stress in the deregulation of processes described above. For a better understanding and comprehension, we decided to classify and display this evidence throughout the multistep carcinogenesis model, considering the pathways involved in tumor initiation, promotion, and progression (Figure 2).

## 3. Particulate Matter-Mediated Oxidative Stress

Particulate matter (PM) is an air pollutant conformed by a heterogeneous mixture of compounds such as carbon, metals, sulfates, nitrates, pollen, endotoxins, bacteria, viruses, and polycyclic aromatic hydrocarbons (PAHs) [64]. The composition of PM is associated with multiple factors: the place of origin, the season of the year, and the emission sources. Evidence of the effect that PM cause at both epidemiological and toxicological levels is concordant [65,66,67,68]. PM is mainly classified according to its aerodynamic diameter. Based on this classification, we recognize three fractions: the coarse fraction (PM_10_), the fine fraction (PM_2.5_), and finally, the ultrafine fraction (PM_0.1_). All fractions are able to enter the respiratory system through inhalation. Once inside of the respiratory tract, PM is deposited in deep regions such as the alveoli, causing effects at the cellular level. Among these effects, the production of oxidative stress stands out [6,69,70,71].

The induction of oxidative stress mediated by PM can be divided into four main mechanisms. Direct PM ROS generation occurs by the presence of free radicals and oxidants on the particle surface, the presence of soluble compounds, the alteration of mitochondrial function/NADPH-oxidases, and finally, the activation of inflammatory cells capable of generating ROS [72,73,74,75]. In PM-mediated oxidative stress, the main soluble components are transition metals followed by PAHs, because their metabolism generates different reactive products [72,76,77]. Transition metals induce the formation of OH^•^ by Fenton-type reactions, while the PAHs lead to ROS production through the quinone redox cycle. The quinone redox cycle involves biotransformation enzymes such as cytochrome P450 and epoxide hydrolase, causing oxidative damage to cellular macromolecules [78]. It is relevant to point out that the combination of both, metallic and organic components included in PM can generate synergies, leading to a higher production of ROS, which destabilizes the antioxidant system inside cells [79]. The importance of transition metals included in PM_10-2.5_ has been clearly described using antioxidants or metal chelators that prevent the effects of PM on oxidative stress [69,80]. Oxidative stress derived from exposure to PM has been directly related to the appearance and exacerbation of multiple diseases, including different types of cancer. Thus, it becomes relevant to define the mechanisms through which PM induce the generation of ROS, which are the main factor involved in its potential for damage [81,82,83].

ROS are generated in a variety of cell models, such as lung epithelial cells and lung macrophages [84,85,86]. Furthermore, phagocytic cells of the innate immune system such as alveolar macrophages (AM) and polymorphonuclear neutrophils (PMN) are characterized by being highly competent producers of ROS, since this mechanism allows to eliminate pathogens and, in this case, potentially harmful particles in a more efficient way [87]. Therefore, both aspects, the excessive ROS formation and the decrease of the antioxidant and detoxification enzymes (SOD, CAT, GT, and GST) induced by PM are strongly implicated in the genotoxic/cytotoxic mechanisms [88]. Although PM can generate multiple oxidized DNA lesions, multiple studies have focused on the determination of guanine (8-OHdG) oxidation. Moreover, the increase in the levels of 8-OHdG has been reported in both, humans exposed to ambient air as well as in animal and cell models exposed to PM collected from highly polluted cities [70,89,90,91]. Figure 2 summarizes the effect of oxidative stress in cells as well as the biological consequences discussed before.

### 3.1. PM-Mediated Oxidative Stress and Cancer Initiation Mechanisms

Before starting to describe the evidence regarding the role of PM-mediated oxidative stress and cancer, we must consider the nature of PM. PM is a complex mixture that includes genotoxic and non-genotoxic carcinogens in its composition. While genotoxic carcinogens induce DNA damage, non-genotoxic carcinogens alter multiple pathways oriented to induce proliferation, cell survival, endocrine modifications, immune suppression, and toxicity/inflammation responses [92].

Cancer initiation involves the alteration of genes implicated in biochemical signaling pathways oriented to trigger cellular proliferation. Chronic proliferation is, by far, the most fundamental feature of cancer cells. Proliferative signals are widely transmitted by growth factors, which are mainly recognized by cell surface receptors containing intracellular tyrosine kinase domains. In this context, multiple pathways are deregulated in cancer cells, including MAP-kinase, phosphoinositide 3-kinase/protein kinase B (PI3K/AKT), and RAS-extracellular signal-regulated kinase ERK [10]. Diverse studies have reported that ROS can activate proliferation signals [93]. Thus, exposure to PM generates important alterations in a variety of proliferation pathways, activating potential mechanisms of carcinogenesis.

Experimental studies have demonstrated that intratracheal administration of low doses of PM_10_ generates alveolitis, followed by cell proliferation in the lungs of exposed rats. In addition, PM_10_ exposure induced high levels of oxidative stress. However, this work did not explore the pathway associated with cell proliferation increasing [94]. Proliferation after PM_2.5_ exposure was also observed in bone marrow-mesenchymal stem cells, an effect accompanied by the increase of pro-inflammatory cytokines [95]. Moreover, ultrafine particles induce proliferation in rat lung epithelial cells through EGF-R kinase activity in an integrin-dependent manner [96]. These studies indicated that the observed effect on proliferation is attributable to PM-mediated ROS production.

Recent approaches reported that, after PM_2.5_ exposure, the increased ROS induced upregulation of extracellular signal regulatory kinase (ERK) and mitogen-activated protein kinase p38 (MAPK) in the testis of exposed rats [97]. In concordance with these findings, it was reported that PM_2.5_ exposure rapidly stimulates ROS generation in human lung endothelial cells, resulting in increased activation of p38 [98]. Exposure to aqueous extracts of PM_2.5_ in human bronchial epithelial cells (HBE) induces oxidative damage and phosphorylation of p38 as well [99].

In addition to p38 phosphorylation, the expression of multiple MAPKs increases during PM exposure, as a direct effect induced by generated ROS. PM_10_ induces the phosphorylation of the EGF tyrosine receptor, as well as the increase of MEK1/2, and ERK1/2. Changes presented in the phosphorylation of these proteins correlated with the concentration of metals present in PM_10_ [100]. PM_2.5_ exposure also induces MAPK/AP-1 cascade activation, contributing to AT1R upregulation in vascular endothelial cells [101]. Importantly, RAW264.7 cells showed an increase in ERK and NF-κB proteins after PM_2.5_ exposure, while the phosphorylation of p38 was prevented by using the scavenger N-acetylcysteine (NAC) [69]. PM_0.1_ exposure of mouse pulmonary microvascular endothelial cells also increased Erk1/2 phosphorylation and up-regulated early growth response -1 factor (Egr-1) [102].

The PI3K/AKT pathway is also induced by PM-mediated oxidative stress. Rats exposed to PM_2.5_ significantly increase the protein levels of PI3K and AKT [103]. HBE cells exposed to PM_2.5_ also present an increase in AKT phosphorylation levels, an effect correlated with the increase of colony formation [104]. In addition, PM_2.5_ exposure in A549, HBE, and human embryonic stem cells (hESC) increases p65 and ERK phosphorylation levels, an event associated with the ROS increasing. The participation of PM-mediated oxidative stress in the induction of these proteins was demonstrated by the pre-treatment of cells with NAC, preventing the phosphorylation of these proteins [105,106,107,108]. Excessive proliferative signaling can also activate a cellular phenotype called senescence, in which the cell cycle stops even when various proliferative molecules are secreted. These molecules include chemokines, cytokines, and growth factors. Interestingly, these molecules have effects on neighboring cells, inducing malignant transformation in a paracrine way. It has been reported that A549 cells exposed to PM_10_ acquire a phenotype-like senescence detected by the increase in β-galactosidase activity [109]. Moreover, human keratinocytes showed characteristics of cellular senescence after exposure to PM_2.5_ [110]. Both studies associated the senescence phenotype with PM-mediated ROS production.

Tumor suppressor genes are recognized as components that block normal cell transformation to cancerous cells. They are responsible for inhibiting the proliferation of damaged cells by two main strategies: arresting the cell cycle through different phases or inducing apoptosis. During cancer development, its evasion is crucial, because it allows cells to acquire unlimited replicative potential. p53, phosphatase and tensin homolog (PTEN), and retinoblastoma protein (Rb) are the most typical tumor suppressor genes that operate as central control nodes within the cellular processes described above. Rb protein represents an important gatekeeper in cancer development, inducing multiple growth-inhibitory signals by sequestering transcription factors, while p53 senses a variety of intracellular alterations to induce cell cycle arrest until the damage is repaired or activates apoptosis pathways when DNA damage is severe [10]. Multiple genetic and epigenetic mechanisms lead to the loss of function of these genes, mainly through DNA mutations, DNA methylation, and downstream, changes in expression levels. Its inactivation has been characterized in different types of cancer and the negative-feedback mechanisms operate in proliferative signaling circuits, for example, the loss function of PTEN amplifies PI3K signaling and promotes tumorigenesis [10].

PM_2.5_ exposure induce downregulation of tumor suppressor genes p53 and RB in human lung epithelial cells. These alterations are regulated by redox-dependent mechanisms, possibly by covalent metals detected in this fraction [111]. PM_2.5_ exposure in mice shows a time-dependent downregulation of PTEN expression levels in the lung and HBE cells [112]. Furthermore, PM_2.5_ downregulates p53 and PTEN in a dose-dependent as well as time-dependent manner after an increase of 8-oxodG levels [104]. Moreover, PM can deregulate epigenetic mechanisms responsible for regulating the expression of multiple genes. In this context, exposure to high levels of PM_2.5_ with elevated concentrations of metals during pregnancy, increase the methylation of different tumor suppressor genes measured in the placenta, including the p53 promoter [113]. Moreover, PM_2.5_ exposure in mice shows an increase in the levels of 8-oxodG in the lungs, as well as an increase in the methylation of the p16 gene promoter, associated with the positive regulation of DNMT1. These results were in concordance with in vitro effects, in which an increase in mitochondrial oxidation and hypermethylation of p16 was observed in lung alveolar cells isolated from mice and exposed to PM_2.5_ [114]. PM_2.5_ exposure inhibits p53 expression in human bronchial epithelial cells (BEAS-2B) by hypermethylation of its promoter, due to an increase in DNMT3B protein levels. Because the expression of DNMT3B plays a key role in the epigenetic silencing of specific genes during the initial phase of carcinogenesis, and the increase in DNMT3B is mediated by the activation of the Akt pathway induced by ROS, these results reflect the influence of PM-mediated oxidative stress in cancer-driver epigenetic changes [115].

Another strategy of tumor suppressor genes to limit cancer cell proliferation is the induction of regulatory signals oriented to induce senescence and apoptosis. While cellular senescence establishes an irreversible cell cycle arrest in response to DNA damage caused by multiple modifications, including oxidative stress, apoptosis depurates the cell population via the death signaling in damaged cells. These actions represent an important cellular brake for the accumulation of cellular alterations with heritable potential. PM exposure induces an increase in β-galactosidase activity, a senescence marker, in A549 cells. This effect is associated with oxidative stress induced by PM [109]. Similarly, immortalized human corneal epithelial cells (HCECs), and human keratinocyte cell lines (HaCaT, HEK001, and NHEKs) exposed to PM_2.5_ showed an increase of β-galactosidase activity, being this effect reverted in the presence of NAC [110,116]. PM_2.5_ exposure in human keratinocyte cell lines also induces cellular senescence through the reduction of methylation in the *p16^INK4A^* promoter, leading to an increase in the expression of p16^INK4A^ probably by the effect of the Aryl hydrocarbon receptor pathway (AhR)-ROS signaling pathway. Interestingly, these effects were significatively diminished in presence of NAC [110].

### 3.2. PM-Mediated Oxidative Stress and Mechanisms Oriented to Cancer Promotion

The acquisition of multiple alterations and the establishment of certain adaptative advantages in cancer cells cannot be explained without one of the most important evolutionary forces, survival. Cancer promotion is the lengthiest stage, but is also the breakpoint to malignant transformations since it is still reversible. In promotion, the actively proliferating cells accumulate, and programmed cell death represents the most important barrier to cancer development. Cell death mechanisms in the context of the disease have been widely studied during the past decades. Currently, we have vast information regarding the signaling cascade that regulates the apoptotic program. A variety of physiologic stress events trigger the apoptosis pathways, such as the elevation of oncogenic signaling, hyperproliferation, and DNA damage. Cancer cells are characterized for harboring a cell death resistance phenotype, acquired through alterations on the components implicated in the apoptotic machinery, such as apoptosis upstream regulators and apoptosis downstream effectors [117]. Cell death resistance is crucial for cancer establishment and progression and usually correlates with the grade of malignancy in tumors. Advanced-stage tumors exhibit a higher cell death-resistance phenotype than early-stage tumors. Moreover, resistance to cancer therapies is reported frequently in patients with advanced-stage tumors due to the inability to induce cytotoxicity and, therefore, induce apoptosis in cancer cells.

Human bronchial epithelial cells (16HBE14o) exposed to PM exhibit an antiapoptotic effect associated with Fe. This effect is enhanced by benzo(a)pyrene, triggering cellular ROS generation, and activation of NRF2. Activation of NRF2 upregulates several target genes, such as HO1, NQO1, and GPX1. Moreover, NRF2 activation modulates genes implicated in cell death control such as BCL2, BAX, and p53. Thus, the iron component has a role in the cell death-resistance phenotype caused by PM through the activation of the NRF2-dependent antioxidant process [118].

Zinc is another component present in PM that has been associated with a cytoprotective effect. Interestingly, zinc is capable of inhibiting apoptosis and minimizing the oxidative stress caused by the component mixture in PM, mainly linked to lipid peroxidation [119]. Zinc directly protects cells through the stabilization of lipids and proteins of cellular and organelle membranes, as well as indirectly causing an antiapoptotic effect through the maintenance of glutathione levels [120]. Moreover, the cell death-resistant phenotype of zinc has been linked to the reduction of DNA fragmentation (a phenotype induced in cells triggering apoptosis), as well as a reduction in the processing of procaspase-3 and the activation of cell survival pathways such as Akt and ERK in airway epithelial cells [121]. These findings become relevant, since the findings suggest an effect of zinc as an antioxidant factor by itself, whereas the mixture in PM (including zinc) plays a role as an oxidant factor.

Additionally, other transition metals present in PM such as magnesium, strontium, and manganese are implicated in the cell death-resistance phenotype. It has been reported that these components can inhibit the calcium-induced permeability transition pore (PTP) opening competitively. Although the precise mechanism is still unknown, these findings suggest a possible protective effect of metallic compounds on mitochondria, preventing permeability and, therefore, the release of multiple pro-apoptotic factors [122].

PM_2.5_ treatment prevents mitochondria-driven apoptosis in cell lines and primary cultures of human bronchial epithelial cells, exhibiting reductions in the frequency of morphologic changes associated with apoptosis such as cell size decrease, chromatin condensation, and formation of apoptotic bodies, an effect associated with PAHs [123]. Furthermore, surviving cells after BaP exposure can form tumors in nude mice, indicating a role of the anti-apoptotic phenotype in cell transformation [124]. The combined effect of BaP and air pollution gases increase the levels of anti-apoptosis proteins and decrease the levels of pro-apoptotic proteins in lung fibroblast cell line MRC-5 [125]. PM_2.5_ exposure also produces an anti-apoptotic effect resulting from both PAHs and transition metals, in an additive fashion. The anti-apoptotic effect of metals was predominant in metals and enhanced by certain PAHs. Iron in PM_2.5_ can activate NRF2, followed by repression of genes involved in cell death pathways [118,126]. Anti-apoptotic signaling has been also attributed to PAHs present in PM_10_ and PM_2.5_, followed by the production of ROS by PAHs metabolism. It appears that PM exposure represents a strong factor that dictates cell fate, with the participation of ROS, PAHs, and metals in the induction of cell death [127].

PM_10_ exposure also promotes cell death resistance when oxidative stress is over-induced. Interestingly, in this work, the challenge with a second oxidant stimulus (H_2_O_2_) induced the evasion of apoptosis via upregulation of PI3K/AKT/FoxO3a in A549 cells. When PI3K function was blocked by a pharmacological inhibitor, apoptosis was remarkably increased when cells were exposed to both stimuli [128]. These results are in concordance with other results, since PM_2.5_ also causes evasion of apoptosis in BEAS-2B cells through the PI3K-AKT pathway [129]. On the opposite, autophagy, another cell-physiologic response involved in apoptosis resistance has been studied in the context of PM exposure. In this context, PM_2.5_ induced autophagy by the inhibition of the PI3K-AKT-mTOR signaling pathway in BEAS-2B cells, while inhibition of autophagy induces apoptosis and cytotoxicity in A549 cells treated by PM_2.5_ [130,131]. Interestingly, UFP exhibits low levels of apoptosis, suggesting that particles may cause impairment of mitochondrial-nuclear crosstalk, causing mitochondrial membrane depolarization, altered mitochondrial respiratory chain enzyme activity, and a reduction in mitochondrial DNA copy number, altering redox homeostasis and inducing cell survival [132]. Exposure to PM_10_ and PM_2.5_ activates the NF-κB promoter and increases the levels and phosphorylation of NF-κB and its DNA binding activity causing an anti-apoptotic effect in A549, BEAS-2B, murine alveolar macrophage RAW 264.7, and human umbilical vein endothelial cells (HUVEC) [126,133,134,135,136,137,138,139]. Experiments using organ-level lung microfluidic systems have reported the induction of the anti-apoptotic pathways in lung epithelial cells [129]. The anti-apoptotic effect PM_10_ and PM_2.5_ induced has been deeply investigated and several signaling pathways including oxidative stress are involved [109,126,137,138,139,140].

Once cancer cells have acquired their capacity to proliferate and inhibit apoptosis independently of external stimulus, the next stage required is the acquisition of unlimited replicative potential. This feature is developed against the common way of many cell lineages since they can accomplish only a limited number of division cycles. Immortalization represents the threshold between cancer initiation and promotion and establishes the conditions for a more aggressive and effective expansion during tumorigenesis. Telomeric regulation represents the central point for unlimited proliferation [141,142]. Telomeres are conformed by multiple tandem hexameric repeats subject to shortening in normal cells, a process usually termed “telomeric erosion”. The function of telomeres is the protection of the ends of chromosomal DNA to form end-to-end fusions with other chromosomes, avoiding the scrambling of karyotype ad preventing cells from developing genomic/chromosomal instability [143]. Because the length of telomeric DNA determines the capability of cells to duplicate, and erosion reduces this capability, telomeric regulation becomes important in cancer. Transformed cells expressed telomerase, a specific DNA polymerase involved in the addition of telomere repeat segments to the ends of telomeric DNA, avoiding senescence and promoting cell immortalization [144]. Together, telomerase and other proteins involved in telomere-regulation induce cell immortalization and, therefore, cancer promotion. Interestingly, replicative immortality by telomeric regulation is a hallmark not induced by PM-mediated oxidative stress. The majority of epidemiological studies point to the fact that exposure to air pollution PM causes telomere length shortening in multiple sources (leukocyte, saliva placental, and buccal cells and sperm) where PM was assessed [145]. However, the role of air pollution in telomeric regulation, and most importantly, the role of PM-mediated oxidative stress in telomere shortening has been linked with alternative pathways oriented to the acquisition of other cancer hallmarks.

PM_2.5_ exposure in urban areas causes telomere shortening in humans, an effect caused by ROS and RNS [146,147]. DNA base damage caused by PM-mediated oxidative stress accumulates in individuals, making them susceptible to an accelerated senescence phenotype. Modification of guanines and 8-oxodG bases increase after PM-mediated oxidative stress is produced by ROS [148,149]. Human telomere shortening and, therefore, loss of protection of chromosomal ends activates RB and p53 pathways to maintain genomic stability and cell functionality. However, as mentioned previously, these pathways are compromised by PM-mediated oxidative stress. Thus, telomere shortening predisposes cells to the generation of genomic instability, leading to chromosomal breaking and chromosomal end-to-end fusions, making them capable of acquiring multiple genetic changes and mutations [150,151]. Telomere maintenance does not have a crucial role in cancer promotion, and telomerase activation (the reverse transcriptase responsible for telomere elongation) promotes carcinogenesis also in an independent way.

### 3.3. PM-Mediated Oxidative Stress and Mechanisms Involved in Cancer Progression

Progression is the final stage of malignant transformation, being the point of no return, where genotypic alterations are widely manifested in the phenotype and morphology of cancer cells. The survival, development, and correct cellular functions of cells resemble the adequate supply of oxygen and nutrients. Therefore, during carcinogenesis, the growth of new vasculature is required. Transformed cells develop angiogenic capabilities that drive the formation of new blood vessels from pre-existing vessels. This process is orchestrated through signals transmitted by soluble factors and their receptors, allowing tumor promotion and progression. Thus, vascular endothelial growth factor (VEGF), acid and basic fibroblast growth factors (FGF), and Platelet-derived growth factor (PDGF) are signals that bind to transmembrane tyrosine kinase receptors of endothelial cells and initiate the angiogenesis, whereas thrombospondin-1 (TSP-1) or β-interferon inhibit this process, keeping an angiogenic equilibrium. In cancer, this equilibrium is lost, leading to the activation of the “angiogenic switch”. Moreover, factors such as intercellular adhesion molecule-1 (ICAM-1), Interleukin-8 (IL-8), TNF-α (Tumor necrosis factor-α), and angiopoietin participate during the new vessel network conformation [10].

Human environmental exposure to high levels of PM_2.5_ is associated with an increase in VEGF levels in the blood, an effect significantly related to markers of oxidative stress [152,153]. Furthermore, in a study of non-smoking humans exposed to concentrated ambient particles (CAP), it was determined that after one hour of exposure to the coarse fraction of CAP (10–2.5 µm), blood VEGF levels increased as a response to PM_10_ exposure, while an increase in urinary VEGF was observed after exposure to CAP fine fraction (2.5–0.15 μm). These changes were correlated with the presence of endotoxins and associated with the generation of increased urinary levels of 8-OHdG in the coarse fraction and of MDA in the fine fraction [154]. On the other hand, the organic fraction derived from PM_2.5_ increases ICAM-1 expression in A549 cells as well as in mice intratracheally instilled through the IL-6/AKT/STAT3/NF-κB signaling pathway [108]. Another study reported that mice that were intrabronchially instilled with PM_2.5_ showed an upregulation of iNOS, IL-1β, and IL-18 genes in lung tissue [155]. In vivo alterations in these pathways are also accompanied by alterations in the levels of DNA damage markers. Male Wistar rats exposed to PM_2.5_ exhibited an increase in DNA repair genes such as OGG1, as well as a decrease in both DNA repair genes MTH1 and XRCC1 [156].

In vitro models have indicated that PM exposure can promote angiogenesis by ROS generation. PM_10_ induces PDGF α-receptor expression in rat lung myofibroblasts through a pathway dependent on macrophage activation involving IL-1β. This change is probably stimulated by the synergistic effects of metals and lipopolysaccharide contained in PM [157]. In a study performed on human lung epithelial cell line (BEAS-2B) grown on microfluidic chips, it was described that PM_2.5_ upregulated both the gene and protein of VEGFA and that the FGF/FGFR/MAPK/VEGF signal pathway was active, maintaining the sustained angiogenesis signal [129]. Moreover, PM exposure disrupts the barrier of pulmonary endothelial cells increasing vascular permeability through the activation of RhoA/ROCK and the disruption of cytoskeleton rearrangement by the inhibition of myosin phosphatase (MYPT). This effect was significantly attenuated in the presence of the scavenger NAC [158].

The most advanced stage during cancer progression is the acquisition of features capable of inducing tumor cell invasion and metastasis. After a series of alterations and the accumulation of adaptative advantages, cancer cells acquire motility (invasion), along with the capacity to induce signals oriented to penetrate the cell barrier surrounding the circulatory and/or lymphatic system (intravasation), and, finally, be subjected to vessel transportation with the potential of colonizing distant tissues (extravasation), where they will develop secondary tumors (metastasis). To reach these sites, cell–cell (cadherins) and cell–matrix (integrins) interactions are modified, to provide an adequate environment for malignant cells to dissociate from the tumor mass and invade the surrounding stroma. In search for the invasive phenotype, cell–cell interactions are altered in malignant cells. Perhaps the most important molecule whose expression is diminished during the invasion process is E-cadherin. E-cadherin is an important adhesion molecule involved in the regulation of antigrowth signals through its intracellular interaction with β-catenin. E-cadherin is a key factor in the process known as the epithelial-mesenchymal transition (EMT), where transcriptional factors such as Snail, Slug, Twist, and Zeb1/2 regulate invasion and metastasis [9,10].

PM_10_ exposure induces EMT through reactive oxygen species-mediated extracellular matrix degradation, causing an increase in metalloproteinases (MMPs) 2 and 9 activity, and the generation of an invasive phenotype in the NCI-H441 cell line. These effects were associated with ROS production since they were attenuated by the chebulic acid antioxidant capacity [159]. Furthermore, human bronchial epithelial cells (HBECs) treated with PM_10_ exhibited an increased expression of MMP-9, an effect caused by ROS and attenuated in the presence of NAC [160]. In addition, PM_10_ exposure decrease the levels of tight junction proteins such as zonula occludins (ZO)-1, occludin, claudin-1, and E-cadherin in fresh inferior turbinate tissue cells as well as lung endothelial cells. These effects were as well associated with ROS production and prevented by NAC, where epithelial integrity was maintained [161,162]. Moreover, PM_10_ exposure destabilizes cell junctions via tyrosine phosphorylation and internalization of vascular endothelial-cadherin (VE-cadherin) in human pulmonary artery endothelial cells, an effect attributed to the formation of products of phospholipid peroxidation [163]. Similar effects were observed for PM_2.5_ exposure, causing cell membrane damage via ROS production and increased vascular permeability in endothelial vein cells (HUVEC). Additionally, PM_2.5_ activates VEGFR2 and its downstream MAPK/ERK signaling, leading to VE-cadherin internalization [164]. Finally, PM_2.5_ exposure induces oxidative stress in lung tissues of Sprague Dawley rats, evidenced by an increase in MDA and a decrease in SOD and GSH enzymes. These effects were associated with EMT up-regulation observed through the TGF-β/Smad3 pathway [165,166]. Because PM represents a heterogeneous mixture, the generation of intracellular ROS is subject to the synergism of redox properties inherent to inorganic and organic components such as metals, PAHs, and volatile organic compounds. Thus, oxidative stress regulates pathways oriented to enhance proliferation, repress apoptosis, and promote angiogenesis and metastasis, all key events during cancer development [167].

## 4. Clues Regarding the Integration of PM-Mediated Oxidative Stress during the Cancer Continuum

Because air pollution has increased in highly populated cities, and we are constantly exposed to this silent risk factor, during the last decades, the impact of environmentally induced oxidative stress on human health caused by pollutants has become relevant [168]. Chronic exposure to environmental sources of oxidative stress results in a loss of the oxidant/antioxidant balance, leading to cellular damage in multiple functions and components. This sustained stimulus contributes to the arising of multiple human diseases such as diabetes, cardiovascular diseases, neurodegenerative diseases, and cancer.

The multistep model of carcinogenesis involves three main stages: initiation, promotion, and progression. In this context, oxidative stress has been considered an important factor for the establishment of every stage. Furthermore, the contribution of oxidative stress during cancer development has been strongly related to a variety of altered signaling pathways involved in crucial cellular events. These events include sustained proliferative signaling, evasion growth suppression, cell death resistance, replicative immortality, angiogenesis, and invasion/metastasis [10,169]. The multistep model of carcinogenesis is hierarchical, as well as the acquisition of cancer adaptative advantages. Figure 2 describes the sequential events associated with PM-mediated oxidative stress during malignant transformation discussed in this approach. Cancer initiation implicates the gain of function for sustained proliferative signaling and the loss of function of growth suppression signaling, representing the first step during malignant transformation. Both features are essential for tumor arising since establish the proper conditions for cancer cell “fitness”. Cancer cells adapt to their environment and expand in a better way than other cell populations. Then, cancer promotion implicates the ability to resist cell death, evading apoptosis. At this point, cancer cells induce multiple signals oriented to survival, making them capable of accumulating alterations and developing genomic instability. Finally, cancer progression implicates the ability of tumor cells to modify the environment, to expand the cancer cell population to sites beyond its immediate surroundings. In this stage, cancer cells induce the formation of blood vessels from a pre-existing network to cover the nutrient/oxygen exchange, accompanied by the ability to colonize distant sites through the metastatic cascade.

ROS interact with cellular biomolecules such as lipids, proteins, and DNA. Chronic alteration of these components conducts the acquisition of a malignant phenotype. ROS promote lipid peroxidation, making them highly reactive and capable of interacting with other membrane phospholipids and causing a sustained chain reaction that stops only by the effect of antioxidants [46,170]. Induction of membrane instability by lipid peroxidation results in the formation of aldehydes, molecules considered the gold standard biomarkers for oxidative stress [171]. In addition, the impact of aldehydes in cellular homeostasis goes beyond membrane instability since they can form adducts with DNA and proteins. Aldehyde-DNA adducts have an active role in carcinogenesis through the induction of sequence frameshift mutations, base-pair substitutions, and the generation of inter-strand cross-links [172]. Moreover, aldehyde-DNA adducts have been considered carcinogenic factors since they can induce G to T transversions inside hotspot sites of the p53 gene sequence, having an impact on many types of cancer [173,174]. These alterations caused by PM-mediated oxidative stress set the conditions for the development of genomic instability, a phenotype frequently observed in transformed cells, where the mechanisms responsible for sensing and repairing DNA damage are compromised, predisposing cells to the acquisition of multiple mutations and chromosomal instability, features constantly observed in tumors and associated with the aggressiveness of the disease.

Oxidative stress represents a physiological/pathological imbalance in cells, representing an important challenge in metabolism since the disruption of redox signaling and the induction of molecular damage becomes a risk factor for malignant transformation [175,176,177]. Together, reactive oxygen species (ROS) affect DNA, lipids, and proteins [177,178,179]. While endogenous ROS formation resembles the metabolism of mitochondria, endoplasmic reticulum, and peroxisomes, processes such as cytochrome p450 metabolism and inflammation are also relevant. Moreover, exogenous ROS formation is associated with environmental factors such as diet, UV radiation, and, importantly, air pollutants add a higher grade of complexity to the understanding of PM-mediated oxidative stress in cancer development.

Several studies have described the mechanisms of PM-induced changes by oxidative stress using in vitro models, as an approach to understanding the effects of PM on the body [180,181,182]. Although important, in vitro studies do not represent entirely the effect of oxidative stress caused by PM on the human body. A more realistic approach to understanding the effect of PM exposure is the use of animal models. Some in vivo studies in rats forced to intense pulmonary activity report an increase in the levels of oxygen sensor hypoxia-inducible factor 1α (HIF1α), as well as lysine-specific demethylase 6A (KDM6A), accompanied by a stimulation of mitochondrial oxidative capacity and biogenesis and a reduction in lactate dehydrogenase activity [183]. Interestingly, an environment with a high concentration of PM is capable of affecting oxidative stress, inflammatory responses, and apoptosis in mouse lungs [184]. Exposure to PM_2.5_ in mice promotes oxidative stress and increases the levels of pro-inflammatory factors [185]. Furthermore, exposure to PM acts as a mediator of oxidative stress, increasing both, the pro-inflammatory response and lipid/protein oxidation products [160,186,187,188], suggesting that PM triggers ROS production, but more important, mitochondrial dysfunction through oxidative stress. Moreover, while a study reports an increase in inflammatory response, damaged mitochondria, and apoptosis seen in rats after 24 h of PM exposure [189], other work reported overexpression of Bcl2 and a reduction in BID expression in the lungs of mice exposed to PM [184]. Together, this evidence highlights the importance of in vivo studies to elucidate the role of PM-mediated oxidative stress during the carcinogenic process.

In addition, we need to consider the effect of cancer treatment in the equation, since therapeutic approaches such as radiotherapy impact in a negative fashion, representing another hit of oxidative stress in a compromised tumorigenic environment [177,178]. Treatment against cancer cells with potential sources of oxidative stress establishes two possible scenarios: the induction of cell death and, therefore, sensitivity to therapy, or the induction of tolerable damage followed by cell adaptation and, therefore, resistance to cancer therapy. Thus, PM-mediated oxidative stress represents an important factor not only in cellular processes such as proliferation, growth suppression, cell death, replicative immortality, angiogenesis, invasion, and metastasis. PM-mediated oxidative stress also acts as an important driving force during cancer initiation, promotion, and progression, displaying a high degree of involvement during the cancer continuum.

## References

[1] Pope C.A., Burnett R.T., Thun M.J., Calle E.E., Krewski D., Ito K., Thurston G.D. (2002). Lung cancer, cardiopulmonary mortality, and long-term exposure to fine particulate air pollution. JAMA.

[2] De Kok T.M., Driece H.A., Hogervorst J.G., Briede J.J. (2006). Toxicological assessment of ambient and traffic-related particulate matter: A review of recent studies. Mutat. Res..

[3] Falcon-Rodriguez C.I., Osornio-Vargas A.R., Sada-Ovalle I., Segura-Medina P. (2016). Aeroparticles, Composition, and Lung Diseases. Front. Immunol..

[4] Turner M.C., Andersen Z.J., Baccarelli A., Diver W.R., Gapstur S.M., Pope C.A., Prada D., Samet J., Thurston G., Cohen A. (2020). Outdoor air pollution and cancer: An overview of the current evidence and public health recommendations. CA Cancer J. Clin..

[5] Pitot H.C. (1993). The molecular biology of carcinogenesis. Cancer.

[6] Brauer M., Avila-Casado C., Fortoul T.I., Vedal S., Stevens B., Churg A. (2001). Air pollution and retained particles in the lung. Environ. Health Perspect..

[7] Castaño-Vinyals G., D’Errico A., Malats N., Kogevinas M. (2004). Biomarkers of exposure to polycyclic aromatic hydrocarbons from environmental air pollution. Occup. Environ. Med..

[8] Manisalidis I., Stavropoulou E., Stavropoulos A., Bezirtzoglou E. (2020). Environmental and Health Impacts of Air Pollution: A Review. Front. Public Health.

[9] Hanahan D., Weinberg R.A. (2000). The hallmarks of cancer. Cell.

[10] Hanahan D., Weinberg R.A. (2011). Hallmarks of cancer: The next generation. Cell.

[11] Barrett J.C. (1993). Mechanisms of Multistep Carcinogenesis and Carcinogen Risk Assessment. Environ. Health Perspect..

[12] Sies H., Berndt C., Jones D.P. (2017). Oxidative Stress. Annu. Rev. Biochem..

[13] Cabello-Verrugio C., Simon F., Trollet C., Santibanez J.F. (2017). Oxidative Stress in Disease and Aging: Mechanisms and Therapies 2016. Oxidative Med. Cell. Longev..

[14] Scandalios J.G. (2005). Oxidative stress: Molecular perception and transduction of signals triggering antioxidant gene defenses. Braz. J. Med. Biol. Res. Rev. Bras. De Pesqui. Med. E Biol..

[15] Kohen R., Nyska A. (2002). Oxidation of biological systems: Oxidative stress phenomena, antioxidants, redox reactions, and methods for their quantification. Toxicol. Pathol..

[16] Genestra M. (2007). Oxyl radicals, redox-sensitive signalling cascades and antioxidants. Cell. Signal..

[17] Czerska M., Mikolajewska K., Zielinski M., Gromadzinska J., Wasowicz W. (2015). Today’s oxidative stress markers. Med. Pr..

[18] Zorov D.B., Juhaszova M., Sollott S.J. (2014). Mitochondrial reactive oxygen species (ROS) and ROS-induced ROS release. Physiol. Rev..

[19] Aruoma O.I., Grootveld M., Bahorun T. (2006). Free radicals in biology and medicine: From inflammation to biotechnology. BioFactors.

[20] Snezhkina A.V., Kudryavtseva A.V., Kardymon O.L., Savvateeva M.V., Melnikova N.V., Krasnov G.S., Dmitriev A.A. (2019). ROS Generation and Antioxidant Defense Systems in Normal and Malignant Cells. Oxidative Med. Cell. Longev..

[21] Marengo B., Nitti M., Furfaro A.L., Colla R., Ciucis C.D., Marinari U.M., Pronzato M.A., Traverso N., Domenicotti C. (2016). Redox Homeostasis and Cellular Antioxidant Systems: Crucial Players in Cancer Growth and Therapy. Oxidative Med. Cell. Longev..

[22] Battelli M.G., Polito L., Bortolotti M., Bolognesi A. (2016). Xanthine Oxidoreductase-Derived Reactive Species: Physiological and Pathological Effects. Oxidative Med. Cell. Longev..

[23] Kaludercic N., Mialet-Perez J., Paolocci N., Parini A., Di Lisa F. (2014). Monoamine oxidases as sources of oxidants in the heart. J. Mol. Cell. Cardiol..

[24] Zhang L., Yu L., Yu C.A. (1998). Generation of superoxide anion by succinate-cytochrome c reductase from bovine heart mitochondria. J. Biol. Chem..

[25] Anilkumar N., Sirker A., Shah A.M. (2009). Redox sensitive signaling pathways in cardiac remodeling, hypertrophy and failure. Front. Biosci. (Landmark Ed.).

[26] Sachse A., Wolf G. (2007). Angiotensin II-induced reactive oxygen species and the kidney. J. Am. Soc. Nephrol..

[27] Packer L., Cadenas E. (2007). Oxidants and antioxidants revisited. New concepts of oxidative stress. Free Radic. Res..

[28] Yukihiro M., Hiramatsu T., Kawano T. (2011). Lethal impacts of cigarette smoke in cultured tobacco cells. Tob. Induc. Dis..

[29] Lakey P.S., Berkemeier T., Tong H., Arangio A.M., Lucas K., Poschl U., Shiraiwa M. (2016). Chemical exposure-response relationship between air pollutants and reactive oxygen species in the human respiratory tract. Sci. Rep..

[30] Yokawa K., Kagenishi T., Baluska F. (2015). UV-B Induced Generation of Reactive Oxygen Species Promotes Formation of BFA-Induced Compartments in Cells of Arabidopsis Root Apices. Front. Plant Sci..

[31] Checa J., Aran J.M. (2020). Reactive Oxygen Species: Drivers of Physiological and Pathological Processes. J. Inflamm. Res..

[32] Neha K., Haider M.R., Pathak A., Yar M.S. (2019). Medicinal prospects of antioxidants: A review. Eur. J. Med. Chem..

[33] Birben E., Sahiner U.M., Sackesen C., Erzurum S., Kalayci O. (2012). Oxidative stress and antioxidant defense. World Allergy Organ. J..

[34] Mattmiller S.A., Carlson B.A., Sordillo L.M. (2013). Regulation of inflammation by selenium and selenoproteins: Impact on eicosanoid biosynthesis. J. Nutr. Sci..

[35] He L., He T., Farrar S., Ji L., Liu T., Ma X. (2017). Antioxidants Maintain Cellular Redox Homeostasis by Elimination of Reactive Oxygen Species. Cell. Physiol. Biochem. Int. J. Exp. Cell. Physiol. Biochem. Pharmacol..

[36] Fridovich I. (1975). Superoxide dismutases. Annu. Rev. Biochem..

[37] Liguori I., Russo G., Curcio F., Bulli G., Aran L., Della-Morte D., Gargiulo G., Testa G., Cacciatore F., Bonaduce D. (2018). Oxidative stress, aging, and diseases. Clin. Interv. Aging.

[38] Sosa V., Moline T., Somoza R., Paciucci R., Kondoh H., ME L.L. (2013). Oxidative stress and cancer: An overview. Ageing Res. Rev..

[39] Sarniak A., Lipinska J., Tytman K., Lipinska S. (2016). Endogenous mechanisms of reactive oxygen species (ROS) generation. Postep. Hig. I Med. Dosw..

[40] Babior B.M. (2000). Phagocytes and oxidative stress. Am. J. Med..

[41] Pisoschi A.M., Pop A. (2015). The role of antioxidants in the chemistry of oxidative stress: A review. Eur. J. Med. Chem..

[42] Mazlumoglu M.R., Ozkan O., Alp H.H., Ozyildirim E., Bingol F., Yoruk O., Kuduban O. (2017). Measuring Oxidative DNA Damage With 8-Hydroxy-2’-Deoxyguanosine Levels in Patients With Laryngeal Cancer. Ann. Otol. Rhinol. Laryngol..

[43] Abolhassani N., Leon J., Sheng Z., Oka S., Hamasaki H., Iwaki T., Nakabeppu Y. (2017). Molecular pathophysiology of impaired glucose metabolism, mitochondrial dysfunction, and oxidative DNA damage in Alzheimer’s disease brain. Mech. Ageing Dev..

[44] Wang Y., Wang W., Wang N., Tall A.R., Tabas I. (2017). Mitochondrial Oxidative Stress Promotes Atherosclerosis and Neutrophil Extracellular Traps in Aged Mice. Arterioscler. Thromb. Vasc. Biol..

[45] Dorszewska J., Kowalska M., Prendecki M., Piekut T., Kozlowska J., Kozubski W. (2021). Oxidative stress factors in Parkinson’s disease. Neural Regen. Res..

[46] Klaunig J.E., Wang Z., Pu X., Zhou S. (2011). Oxidative stress and oxidative damage in chemical carcinogenesis. Toxicol. Appl. Pharmacol..

[47] Moloney J.N., Cotter T.G. (2018). ROS signalling in the biology of cancer. Semin. Cell Dev. Biol..

[48] Ghanbari Movahed Z., Rastegari-Pouyani M., Mohammadi M.H., Mansouri K. (2019). Cancer cells change their glucose metabolism to overcome increased ROS: One step from cancer cell to cancer stem cell?. Biomed. Pharmacother. Biomed. Pharmacother..

[49] Rodic S., Vincent M.D. (2018). Reactive oxygen species (ROS) are a key determinant of cancer’s metabolic phenotype. Int. J. Cancer.

[50] Lyras L., Perry R.H., Perry E.K., Ince P.G., Jenner A., Jenner P., Halliwell B. (1998). Oxidative damage to proteins, lipids, and DNA in cortical brain regions from patients with dementia with Lewy bodies. J. Neurochem..

[51] Fridlich R., Annamalai D., Roy R., Bernheim G., Powell S.N. (2015). BRCA1 and BRCA2 protect against oxidative DNA damage converted into double-strand breaks during DNA replication. DNA Repair.

[52] Lloyd D.R., Phillips D.H. (1999). Oxidative DNA damage mediated by copper(II), iron(II) and nickel(II) fenton reactions: Evidence for site-specific mechanisms in the formation of double-strand breaks, 8-hydroxydeoxyguanosine and putative intrastrand cross-links. Mutat. Res..

[53] Benhar M., Engelberg D., Levitzki A. (2002). ROS, stress-activated kinases and stress signaling in cancer. EMBO Rep..

[54] Valavanidis A., Vlachogianni T., Fiotakis C. (2009). 8-hydroxy-2’ -deoxyguanosine (8-OHdG): A critical biomarker of oxidative stress and carcinogenesis. J. Environ. Sci. Health Part C Environ. Carcinog. Ecotoxicol. Rev..

[55] Sassa A., Odagiri M. (2020). Understanding the sequence and structural context effects in oxidative DNA damage repair. DNA Repair.

[56] Klaunig J.E., Kamendulis L.M. (2004). The role of oxidative stress in carcinogenesis. Annu. Rev. Pharmacol. Toxicol..

[57] Toyokuni S. (2006). Novel aspects of oxidative stress-associated carcinogenesis. Antioxid. Redox Signal..

[58] Tzortzaki E.G., Dimakou K., Neofytou E., Tsikritsaki K., Samara K., Avgousti M., Amargianitakis V., Gousiou A., Menikou S., Siafakas N.M. (2012). Oxidative DNA damage and somatic mutations: A link to the molecular pathogenesis of chronic inflammatory airway diseases. Chest.

[59] Lee D.H., O’Connor T.R., Pfeifer G.P. (2002). Oxidative DNA damage induced by copper and hydrogen peroxide promotes CG-->TT tandem mutations at methylated CpG dinucleotides in nucleotide excision repair-deficient cells. Nucleic Acids Res..

[60] Rahal A., Kumar A., Singh V., Yadav B., Tiwari R., Chakraborty S., Dhama K. (2014). Oxidative stress, prooxidants, and antioxidants: The interplay. BioMed Res. Int..

[61] Gruber D.R., Toner J.J., Miears H.L., Shernyukov A.V., Kiryutin A.S., Lomzov A.A., Endutkin A.V., Grin I.R., Petrova D.V., Kupryushkin M.S. (2018). Oxidative damage to epigenetically methylated sites affects DNA stability, dynamics and enzymatic demethylation. Nucleic Acids Res..

[62] Jin Jung K., Hyun Kim D., Kyeong Lee E., Woo Song C., Pal Yu B., Young Chung H. (2013). Oxidative stress induces inactivation of protein phosphatase 2A, promoting proinflammatory NF-kappaB in aged rat kidney. Free Radic. Biol. Med..

[63] Wiemer E.A. (2011). Stressed tumor cell, chemosensitized cancer. Nat. Med..

[64] Chirino Y.I., Sanchez-Perez Y., Osornio-Vargas A.R., Rosas I., Garcia-Cuellar C.M. (2015). Sampling and composition of airborne particulate matter (PM10) from two locations of Mexico City. Data Brief.

[65] Badyda A.J., Grellier J., Dabrowiecki P. (2016). Ambient PM2.5 Exposure and Mortality Due to Lung Cancer and Cardiopulmonary Diseases in Polish Cities. Adv. Exp. Med. Biol..

[66] Sevastyanova O., Novakova Z., Hanzalova K., Binkova B., Sram R.J., Topinka J. (2008). Temporal variation in the genotoxic potential of urban air particulate matter. Mutat. Res..

[67] Bonetta S., Gianotti V., Bonetta S., Gosetti F., Oddone M., Gennaro M.C., Carraro E. (2009). DNA damage in A549 cells exposed to different extracts of PM(2.5) from industrial, urban and highway sites. Chemosphere.

[68] Raaschou-Nielsen O., Beelen R., Wang M., Hoek G., Andersen Z.J., Hoffmann B., Stafoggia M., Samoli E., Weinmayr G., Dimakopoulou K. (2016). Particulate matter air pollution components and risk for lung cancer. Environ. Int..

[69] He M., Ichinose T., Yoshida S., Ito T., He C., Yoshida Y., Arashidani K., Takano H., Sun G., Shibamoto T. (2017). PM2.5-induced lung inflammation in mice: Differences of inflammatory response in macrophages and type II alveolar cells. J. Appl. Toxicol. JAT.

[70] Knaapen A.M., Schins R.P., Steinfartz Y., Doris H., Dunemann L., Borm P.J. (2000). Ambient Particulate Matter Induces Oxidative Dna Damage in Lung Epithelial Cells. Inhal. Toxicol..

[71] Pope C.A., Dockery D.W. (2006). Health effects of fine particulate air pollution: Lines that connect. J. Air Waste Manag. Assoc..

[72] Li N., Sioutas C., Cho A., Schmitz D., Misra C., Sempf J., Wang M., Oberley T., Froines J., Nel A. (2003). Ultrafine particulate pollutants induce oxidative stress and mitochondrial damage. Environ. Health Perspect..

[73] Schins R.P., Lightbody J.H., Borm P.J., Shi T., Donaldson K., Stone V. (2004). Inflammatory effects of coarse and fine particulate matter in relation to chemical and biological constituents. Toxicol. Appl. Pharmacol..

[74] Risom L., Moller P., Loft S. (2005). Oxidative stress-induced DNA damage by particulate air pollution. Mutat. Res..

[75] Wang Y., Zhang M., Li Z., Yue J., Xu M., Zhang Y., Yung K.K.L., Li R. (2019). Fine particulate matter induces mitochondrial dysfunction and oxidative stress in human SH-SY5Y cells. Chemosphere.

[76] Nel A.E., Diaz-Sanchez D., Li N. (2001). The role of particulate pollutants in pulmonary inflammation and asthma: Evidence for the involvement of organic chemicals and oxidative stress. Curr. Opin. Pulm. Med..

[77] Shi T., Knaapen A.M., Begerow J., Birmili W., Borm P.J., Schins R.P. (2003). Temporal variation of hydroxyl radical generation and 8-hydroxy-2’-deoxyguanosine formation by coarse and fine particulate matter. Occup. Environ. Med..

[78] Bolton J.L., Dunlap T. (2017). Formation and Biological Targets of Quinones: Cytotoxic versus Cytoprotective Effects. Chem. Res. Toxicol..

[79] Lepers C., Andre V., Dergham M., Billet S., Verdin A., Garcon G., Dewaele D., Cazier F., Sichel F., Shirali P. (2014). Xenobiotic metabolism induction and bulky DNA adducts generated by particulate matter pollution in BEAS-2B cell line: Geographical and seasonal influence. J. Appl. Toxicol. JAT.

[80] Son E.S., Park J.W., Kim Y.J., Jeong S.H., Hong J.H., Kim S.H., Kyung S.Y. (2020). Effects of antioxidants on oxidative stress and inflammatory responses of human bronchial epithelial cells exposed to particulate matter and cigarette smoke extract. Toxicol. In Vitro Int. J. Publ. Assoc. BIBRA.

[81] Gurgueira S.A., Lawrence J., Coull B., Murthy G.G., Gonzalez-Flecha B. (2002). Rapid increases in the steady-state concentration of reactive oxygen species in the lungs and heart after particulate air pollution inhalation. Environ. Health Perspect..

[82] Knaapen A.M., Borm P.J., Albrecht C., Schins R.P. (2004). Inhaled particles and lung cancer. Part A: Mechanisms. Int. J. Cancer.

[83] Dergham M., Lepers C., Verdin A., Billet S., Cazier F., Courcot D., Shirali P., Garcon G. (2012). Prooxidant and proinflammatory potency of air pollution particulate matter (PM2.5-0.3) produced in rural, urban, or industrial surroundings in human bronchial epithelial cells (BEAS-2B). Chem. Res. Toxicol..

[84] Jayawardena T.U., Sanjeewa K.K.A., Lee H.G., Nagahawatta D.P., Yang H.W., Kang M.C., Jeon Y.J. (2020). Particulate Matter-Induced Inflammation/Oxidative Stress in Macrophages: Fucosterol from Padina boryana as a Potent Protector, Activated via NF-kappaB/MAPK Pathways and Nrf2/HO-1 Involvement. Mar. Drugs.

[85] Ohyama M., Otake T., Adachi S., Kobayashi T., Morinaga K. (2007). A comparison of the production of reactive oxygen species by suspended particulate matter and diesel exhaust particles with macrophages. Inhal. Toxicol..

[86] den Hartigh L.J., Lame M.W., Ham W., Kleeman M.J., Tablin F., Wilson D.W. (2010). Endotoxin and polycyclic aromatic hydrocarbons in ambient fine particulate matter from Fresno, California initiate human monocyte inflammatory responses mediated by reactive oxygen species. Toxicol. In Vitro Int. J. Publ. Assoc. BIBRA.

[87] Yang Y., Bazhin A.V., Werner J., Karakhanova S. (2013). Reactive oxygen species in the immune system. Int. Rev. Immunol..

[88] Chirino Y.I., Sanchez-Perez Y., Osornio-Vargas A.R., Morales-Barcenas R., Gutierrez-Ruiz M.C., Segura-Garcia Y., Rosas I., Pedraza-Chaverri J., Garcia-Cuellar C.M. (2010). PM(10) impairs the antioxidant defense system and exacerbates oxidative stress driven cell death. Toxicol. Lett..

[89] Kim J.Y., Mukherjee S., Ngo L.C., Christiani D.C. (2004). Urinary 8-hydroxy-2’-deoxyguanosine as a biomarker of oxidative DNA damage in workers exposed to fine particulates. Environ. Health Perspect..

[90] Bagryantseva Y., Novotna B., Rossner P., Chvatalova I., Milcova A., Svecova V., Lnenickova Z., Solansky I., Sram R.J. (2010). Oxidative damage to biological macromolecules in Prague bus drivers and garagemen: Impact of air pollution and genetic polymorphisms. Toxicol. Lett..

[91] Rao X., Zhong J., Brook R.D., Rajagopalan S. (2018). Effect of Particulate Matter Air Pollution on Cardiovascular Oxidative Stress Pathways. Antioxid. Redox Signal..

[92] Hernandez L.G., van Steeg H., Luijten M., van Benthem J. (2009). Mechanisms of non-genotoxic carcinogens and importance of a weight of evidence approach. Mutat. Res..

[93] Zhang J., Wang X., Vikash V., Ye Q., Wu D., Liu Y., Dong W. (2016). ROS and ROS-Mediated Cellular Signaling. Oxidative Med. Cell. Longev..

[94] Gerlofs-Nijland M.E., Dormans J.A., Bloemen H.J., Leseman D.L., John A., Boere F., Kelly F.J., Mudway I.S., Jimenez A.A., Donaldson K. (2007). Toxicity of coarse and fine particulate matter from sites with contrasting traffic profiles. Inhal. Toxicol..

[95] Abu-Elmagd M., Alghamdi M.A., Shamy M., Khoder M.I., Costa M., Assidi M., Kadam R., Alsehli H., Gari M., Pushparaj P.N. (2017). Evaluation of the Effects of Airborne Particulate Matter on Bone Marrow-Mesenchymal Stem Cells (BM-MSCs): Cellular, Molecular and Systems Biological Approaches. Int. J. Environ. Res. Public Health.

[96] Sydlik U., Bierhals K., Soufi M., Abel J., Schins R.P., Unfried K. (2006). Ultrafine carbon particles induce apoptosis and proliferation in rat lung epithelial cells via specific signaling pathways both using EGF-R. Am. J. Physiology. Lung Cell. Mol. Physiol..

[97] Liu B., Wu S.D., Shen L.J., Zhao T.X., Wei Y., Tang X.L., Long C.L., Zhou Y., He D.W., Lin T. (2019). Spermatogenesis dysfunction induced by PM2.5 from automobile exhaust via the ROS-mediated MAPK signaling pathway. Ecotoxicol. Environ. Saf..

[98] Wang T., Chiang E.T., Moreno-Vinasco L., Lang G.D., Pendyala S., Samet J.M., Geyh A.S., Breysse P.N., Chillrud S.N., Natarajan V. (2010). Particulate matter disrupts human lung endothelial barrier integrity via ROS- and p38 MAPK-dependent pathways. Am. J. Respir. Cell Mol. Biol..

[99] Xin L., Che B., Zhai B., Luo Q., Zhang C., Wang J., Wang S., Fan G., Liu Z., Feng J. (2019). 1,25-Dihydroxy Vitamin D3 Attenuates the Oxidative Stress-Mediated Inflammation Induced by PM2.5via the p38/NF-kappaB/NLRP3 Pathway. Inflammation.

[100] Wu W., Samet J.M., Ghio A.J., Devlin R.B. (2001). Activation of the EGF receptor signaling pathway in airway epithelial cells exposed to Utah Valley PM. Am. J. Physiol. Lung Cell. Mol. Physiol..

[101] Xu X., Xu H., Qimuge A., Liu S., Wang H., Hu M., Song L. (2019). MAPK/AP-1 pathway activation mediates AT1R upregulation and vascular endothelial cells dysfunction under PM2.5 exposure. Ecotoxicol. Environ. Saf..

[102] Mo Y., Wan R., Feng L., Chien S., Tollerud D.J., Zhang Q. (2012). Combination effects of cigarette smoke extract and ambient ultrafine particles on endothelial cells. Toxicol. In Vitro Int. J. Publ. Assoc. BIBRA.

[103] Su R., Jin X., Zhang W., Li Z., Liu X., Ren J. (2017). Particulate matter exposure induces the autophagy of macrophages via oxidative stress-mediated PI3K/AKT/mTOR pathway. Chemosphere.

[104] Chen S., Li D., Zhang H., Yu D., Chen R., Zhang B., Tan Y., Niu Y., Duan H., Mai B. (2019). The development of a cell-based model for the assessment of carcinogenic potential upon long-term PM2.5 exposure. Environ. Int..

[105] Deng X., Zhang F., Rui W., Long F., Wang L., Feng Z., Chen D., Ding W. (2013). PM2.5-induced oxidative stress triggers autophagy in human lung epithelial A549 cells. Toxicol. In Vitro Int. J. Publ. Assoc. BIBRA.

[106] Bi S., Tang J., Zhang L., Huang L., Chen J., Wang Z., Chen D., Du L. (2020). Fine particulate matter reduces the pluripotency and proliferation of human embryonic stem cells through ROS induced AKT and ERK signaling pathway. Reprod. Toxicol..

[107] Wang J., Zhu M., Wang L., Chen C., Song Y. (2019). Amphiregulin potentiates airway inflammation and mucus hypersecretion induced by urban particulate matter via the EGFR-PI3Kalpha-AKT/ERK pathway. Cell. Signal..

[108] Liu C.W., Lee T.L., Chen Y.C., Liang C.J., Wang S.H., Lue J.H., Tsai J.S., Lee S.W., Chen S.H., Yang Y.F. (2018). PM2.5-induced oxidative stress increases intercellular adhesion molecule-1 expression in lung epithelial cells through the IL-6/AKT/STAT3/NF-kappaB-dependent pathway. Part. Fibre Toxicol..

[109] Sanchez-Perez Y., Chirino Y.I., Osornio-Vargas A.R., Herrera L.A., Morales-Barcenas R., Lopez-Saavedra A., Gonzalez-Ramirez I., Miranda J., Garcia-Cuellar C.M. (2014). Cytoplasmic p21(CIP1/WAF1), ERK1/2 activation, and cytoskeletal remodeling are associated with the senescence-like phenotype after airborne particulate matter (PM(10)) exposure in lung cells. Toxicol. Lett..

[110] Ryu Y.S., Kang K.A., Piao M.J., Ahn M.J., Yi J.M., Bossis G., Hyun Y.M., Park C.O., Hyun J.W. (2019). Particulate matter-induced senescence of skin keratinocytes involves oxidative stress-dependent epigenetic modifications. Exp. Mol. Med..

[111] Abbas I., Garcon G., Saint-Georges F., Billet S., Verdin A., Gosset P., Mulliez P., Shirali P. (2010). Occurrence of molecular abnormalities of cell cycle in L132 cells after in vitro short-term exposure to air pollution PM(2.5). Chem.-Biol. Interact..

[112] Chao X., Yi L., Lan L.L., Wei H.Y., Wei D. (2020). Long-term PM2.5 exposure increases the risk of non-small cell lung cancer (NSCLC) progression by enhancing interleukin-17a (IL-17a)-regulated proliferation and metastasis. Aging.

[113] Neven K.Y., Saenen N.D., Tarantini L., Janssen B.G., Lefebvre W., Vanpoucke C., Bollati V., Nawrot T.S. (2018). Placental promoter methylation of DNA repair genes and prenatal exposure to particulate air pollution: An ENVIRONAGE cohort study. Lancet Planet. Health.

[114] Soberanes S., Gonzalez A., Urich D., Chiarella S.E., Radigan K.A., Osornio-Vargas A., Joseph J., Kalyanaraman B., Ridge K.M., Chandel N.S. (2012). Particulate matter Air Pollution induces hypermethylation of the p16 promoter Via a mitochondrial ROS-JNK-DNMT1 pathway. Sci. Rep..

[115] Zhou W., Tian D., He J., Wang Y., Zhang L., Cui L., Jia L., Zhang L., Li L., Shu Y. (2016). Repeated PM2.5 exposure inhibits BEAS-2B cell P53 expression through ROS-Akt-DNMT3B pathway-mediated promoter hypermethylation. Oncotarget.

[116] Gao Z.X., Song X.L., Li S.S., Lai X.R., Yang Y.L., Yang G., Li Z.J., Cui Y.H., Pan H.W. (2016). Assessment of DNA Damage and Cell Senescence in Corneal Epithelial Cells Exposed to Airborne Particulate Matter (PM2.5) Collected in Guangzhou, China. Investig. Ophthalmol. Vis. Sci..

[117] D’Arcy M.S. (2019). Cell death: A review of the major forms of apoptosis, necrosis and autophagy. Cell Biol. Int..

[118] Lovera-Leroux M., Crobeddu B., Kassis N., Petit P.X., Janel N., Baeza-Squiban A., Andreau K. (2015). The iron component of particulate matter is antiapoptotic: A clue to the development of lung cancer after exposure to atmospheric pollutants?. Biochimie.

[119] Truong-Tran A.Q., Carter J., Ruffin R.E., Zalewski P.D. (2001). The role of zinc in caspase activation and apoptotic cell death. Biometals.

[120] Szuster-Ciesielska A., Plewka K., Daniluk J., Kandefer-Szerszeń M. (2008). Zinc inhibits ethanol-induced HepG2 cell apoptosis. Toxicol. Appl. Pharmacol..

[121] Carter J.E., Truong-Tran A.Q., Grosser D., Ho L., Ruffin R.E., Zalewski P.D. (2002). Involvement of redox events in caspase activation in zinc-depleted airway epithelial cells. Biochem. Biophys. Res. Commun..

[122] Bernardi P., Krauskopf A., Basso E., Petronilli V., Blalchy-Dyson E., Di Lisa F., Forte M.A. (2006). The mitochondrial permeability transition from in vitro artifact to disease target. FEBS J..

[123] Ferecatu I., Borot M.C., Bossard C., Leroux M., Boggetto N., Marano F., Baeza-Squiban A., Andreau K. (2010). Polycyclic aromatic hydrocarbon components contribute to the mitochondria-antiapoptotic effect of fine particulate matter on human bronchial epithelial cells via the aryl hydrocarbon receptor. Part. Fibre Toxicol..

[124] Teranishi M., Toyooka T., Ohura T., Masuda S., Ibuki Y. (2010). Benzo[a]pyrene exposed to solar-simulated light inhibits apoptosis and augments carcinogenicity. Chem.-Biol. Interact..

[125] Chen J.H., Chou F.P., Lin H.H., Wang C.J. (2005). Gaseous nitrogen oxide repressed benzo[a]pyrene-induced human lung fibroblast cell apoptosis via inhibiting JNK1 signals. Arch. Toxicol..

[126] Shang Y., Zhou Q., Wang T., Jiang Y., Zhong Y., Qian G., Zhu T., Qiu X., An J. (2017). Airborne nitro-PAHs induce Nrf2/ARE defense system against oxidative stress and promote inflammatory process by activating PI3K/Akt pathway in A549 cells. Toxicol. In Vitro Int. J. Publ. Assoc. BIBRA.

[127] Peixoto M.S., de Oliveira Galvao M.F., Batistuzzo de Medeiros S.R. (2017). Cell death pathways of particulate matter toxicity. Chemosphere.

[128] García-Cuellar C.M., Chirino Y.I., Morales-Bárcenas R., Soto-Reyes E., Quintana-Belmares R., Santibáñez-Andrade M., Sánchez-Pérez Y., García-Cuellar C.M., Chirino Y.I., Morales-Bárcenas R. (2020). Airborne particulate matter (PM_10_) inhibits apoptosis through PI3K/AKT/FoxO_3_a pathway in lung epithelial cells: The role of a second oxidant stimulus. Int. J. Mol. Sci..

[129] Zheng L., Liu S., Zhuang G., Xu J., Liu Q., Zhang X., Deng C., Guo Z., Zhao W., Liu T. (2017). Signal Transductions of BEAS-2B Cells in Response to Carcinogenic PM2.5 Exposure Based on a Microfluidic System. Anal. Chem..

[130] Liu T., Wu B., Wang Y., He H., Lin Z., Tan J., Yang L., Kamp D.W., Zhou X., Tang J. (2015). Particulate matter 2.5 induces autophagy via inhibition of the phosphatidylinositol 3-kinase/Akt/mammalian target of rapamycin kinase signaling pathway in human bronchial epithelial cells. Mol. Med. Rep..

[131] Deng X., Zhang F., Wang L., Rui W., Long F., Zhao Y., Chen D., Ding W. (2014). Airborne fine particulate matter induces multiple cell death pathways in human lung epithelial cells. Apoptosis Int. J. Program. Cell Death.

[132] Bhargava A., Tamrakar S., Aglawe A., Lad H., Srivastava R.K., Mishra D.K., Tiwari R., Chaudhury K., Goryacheva I.Y., Mishra P.K. (2018). Ultrafine particulate matter impairs mitochondrial redox homeostasis and activates phosphatidylinositol 3-kinase mediated DNA damage responses in lymphocytes. Environ. Pollut..

[133] Kafoury R.M., Madden M.C. (2005). Diesel exhaust particles induce the over expression of tumor necrosis factor-alpha (TNF-alpha) gene in alveolar macrophages and failed to induce apoptosis through activation of nuclear factor-kappaB (NF-kappaB). Int. J. Environ. Res. Public Health.

[134] Arenz A., Hellweg C.E., Stojicic N., Baumstark-Khan C., Grotheer H.H. (2006). Gene expression modulation in A549 human lung cells in response to combustion-generated nano-sized particles. Ann. N. Y. Acad. Sci..

[135] Montiel-Davalos A., Ibarra-Sanchez Mde J., Ventura-Gallegos J.L., Alfaro-Moreno E., Lopez-Marure R. (2010). Oxidative stress and apoptosis are induced in human endothelial cells exposed to urban particulate matter. Toxicol. In Vitro Int. J. Publ. Assoc. BIBRA.

[136] Bourgeois B., Owens J.W. (2014). The influence of Hurricanes Katrina and Rita on the inflammatory cytokine response and protein expression in A549 cells exposed to PM2.5 collected in the Baton Rouge-Port Allen industrial corridor of Southeastern Louisiana in 2005. Toxicol. Mech. Methods.

[137] Wan Q., Yang Y.P., Liu Z.Y. (2016). Study of Berberine on Attenuating PM2.5-Induced Vascular Endothelial Cells Injury by ERK1/2 Signal Pathway. Zhong Yao Cai Zhongyaocai J. Chin. Med. Mater..

[138] Wan Q., Yang Y.P., Liu Z.Y. (2016). Puerarin attenuates PM2.5-induced vascular endothelial cells injury via ERK1/2 signaling pathway. Zhongguo Zhong Yao Za Zhi Zhongguo Zhongyao Zazhi China J. Chin. Mater. Med..

[139] Tas I., Zhou R., Park S.Y., Yang Y., Gamage C.D.B., Son Y.J., Paik M.J., Kim H. (2019). Inflammatory and tumorigenic effects of environmental pollutants found in particulate matter on lung epithelial cells. Toxicol. In Vitro Int. J. Publ. Assoc. BIBRA.

[140] Reyes-Zarate E., Sanchez-Perez Y., Gutierrez-Ruiz M.C., Chirino Y.I., Osornio-Vargas A.R., Morales-Barcenas R., Souza-Arroyo V., Garcia-Cuellar C.M. (2016). Atmospheric particulate matter (PM10) exposure-induced cell cycle arrest and apoptosis evasion through STAT3 activation via PKCzeta and Src kinases in lung cells. Environ. Pollut..

[141] Blasco M.A. (2005). Telomeres and human disease: Ageing, cancer and beyond. Nat. Rev. Genet..

[142] Shay J.W., Wright W.E. (2000). Hayflick, his limit, and cellular ageing. Nat. Reviews. Mol. Cell Biol..

[143] Blackburn E.H. (1991). Structure and function of telomeres. Nature.

[144] Blackburn E.H. (2001). Switching and signaling at the telomere. Cell.

[145] Zhao B., Vo H.Q., Johnston F.H., Negishi K.J.C.D. (2018). Therapy. Air pollution and telomere length: A systematic review of 12,058 subjects. Cardiovasc. Diagn. Ther..

[146] Hou L., Wang S., Dou C., Zhang X., Yu Y., Zheng Y., Avula U., Hoxha M., Díaz A., McCracken J. (2012). Air pollution exposure and telomere length in highly exposed subjects in Beijing, China: A repeated-measure study. Environ. Int..

[147] Grahame T.J., Schlesinger R.B. (2012). Oxidative stress-induced telomeric erosion as a mechanism underlying airborne particulate matter-related cardiovascular disease. Part. Fibre Toxicol..

[148] von Zglinicki T., Martin-Ruiz C.M., Saretzki G. (2005). Telomeres, cell senescence and human ageing. Signal Transduct..

[149] Zglinicki T.v., Martin-Ruiz C.M. (2005). Telomeres as Biomarkers for Ageing and Age-Related Diseases. Curr. Mol. Med..

[150] De Lange T. (2005). Telomere-related genome instability in cancer. Cold Spring Harb. Symp. Quant. Biol..

[151] Prescott J., Wentzensen I.M., Savage S.A., De Vivo I. (2012). Epidemiologic evidence for a role of telomere dysfunction in cancer etiology. Mutat. Res./Fundam. Mol. Mech. Mutagen..

[152] Liu L., Ruddy T., Dalipaj M., Poon R., Szyszkowicz M., You H., Dales R.E., Wheeler A.J. (2009). Effects of indoor, outdoor, and personal exposure to particulate air pollution on cardiovascular physiology and systemic mediators in seniors. J. Occup. Environ. Med..

[153] Riggs D.W., Zafar N., Krishnasamy S., Yeager R., Rai S.N., Bhatnagar A., O’Toole T.E. (2020). Exposure to airborne fine particulate matter is associated with impaired endothelial function and biomarkers of oxidative stress and inflammation. Environ. Res..

[154] Liu L., Urch B., Poon R., Szyszkowicz M., Speck M., Gold D.R., Wheeler A.J., Scott J.A., Brook J.R., Thorne P.S. (2015). Effects of ambient coarse, fine, and ultrafine particles and their biological constituents on systemic biomarkers: A controlled human exposure study. Environ. Health Perspect..

[155] Jin Y., Wu W., Zhang W., Zhao Y., Wu Y., Ge G., Ba Y., Guo Q., Gao T., Chi X. (2017). Involvement of EGF receptor signaling and NLRP12 inflammasome in fine particulate matter-induced lung inflammation in mice. Environ. Toxicol..

[156] Li R., Zhao L., Zhang L., Chen M., Shi J., Dong C., Cai Z. (2017). Effects of ambient PM(2.5) and 9-nitroanthracene on DNA damage and repair, oxidative stress and metabolic enzymes in the lungs of rats. Toxicol. Res. (Camb.).

[157] Bonner J.C., Rice A.B., Lindroos P.M., O’Brien P.O., Dreher K.L., Rosas I., Alfaro-Moreno E., Osornio-Vargas A.R. (1998). Induction of the lung myofibroblast PDGF receptor system by urban ambient particles from Mexico City. Am. J. Respir. Cell Mol. Biol..

[158] Wang T., Shimizu Y., Wu X., Kelly G.T., Xu X., Wang L., Qian Z., Chen Y., Garcia J.G.N. (2017). Particulate matter disrupts human lung endothelial cell barrier integrity via Rho-dependent pathways. Pulm. Circ..

[159] Lee H.R., Pyo M.C., Chae S.A., Hong C.O., Lee K.W. (2019). Inhibitory Effect of Chebulic Acid on Alveolar Epithelial to Mesenchymal Transition in Response to Urban Particulate Matter Using Co-treatment and Post-treatment Exposure. Biol. Pharm. Bull..

[160] Wang J., Huang J., Wang L., Chen C., Yang D., Jin M., Bai C., Song Y. (2017). Urban particulate matter triggers lung inflammation via the ROS-MAPK-NF-κB signaling pathway. J. Thorac. Dis..

[161] Lee D.C., Choi H., Oh J.M., Lee J., Lee H.Y., Kang J.Y. (2020). Urban particulate matter regulates tight junction proteins by inducing oxidative stress via the Akt signal pathway in human nasal epithelial cells. Toxicol. Lett..

[162] Wang T., Wang L., Moreno-Vinasco L., Lang G.D., Siegler J.H., Mathew B., Usatyuk P.V., Samet J.M., Geyh A.S., Breysse P.N. (2012). Particulate matter air pollution disrupts endothelial cell barrier via calpain-mediated tight junction protein degradation. Part. Fibre Toxicol..

[163] Karki P., Meliton A., Shah A., Tian Y., Ohmura T., Sarich N., Birukova A.A., Birukov K.G. (2018). Role of truncated oxidized phospholipids in acute endothelial barrier dysfunction caused by particulate matter. PLoS ONE.

[164] Long Y.M., Yang X.Z., Yang Q.Q., Clermont A.C., Yin Y.G., Liu G.L., Hu L.G., Liu Q., Zhou Q.F., Liu Q.S. (2020). PM2.5 induces vascular permeability increase through activating MAPK/ERK signaling pathway and ROS generation. J. Hazard. Mater..

[165] Tang W., Du L., Sun W., Yu Z., He F., Chen J., Li X., Yu L., Chen D. (2017). Maternal exposure to fine particulate air pollution induces epithelial-to-mesenchymal transition resulting in postnatal pulmonary dysfunction mediated by transforming growth factor-β/Smad3 signaling. Toxicol. Lett..

[166] Sun B., Shi Y., Li Y., Jiang J., Liang S., Duan J., Sun Z. (2020). Short-term PM. J. Hazard. Mater..

[167] Lee C.-W., Vo T.T.T., Wu C.-Z., Chi M.-C., Lin C.-M., Fang M.-L., Lee I.-T. (2020). The Inducible Role of Ambient Particulate Matter in Cancer Progression via Oxidative Stress-Mediated Reactive Oxygen Species Pathways: A Recent Perception. Cancers.

[168] Lushchak V.I. (2014). Free radicals, reactive oxygen species, oxidative stress and its classification. Chem.-Biol. Interact..

[169] Sies H., Jones D.P. (2020). Reactive oxygen species (ROS) as pleiotropic physiological signalling agents. Nat. Rev. Mol. Cell Biol..

[170] Hayes J.D., Dinkova-Kostova A.T., Tew K.D. (2020). Oxidative Stress in Cancer. Cancer Cell.

[171] Barrera G., Pizzimenti S., Daga M., Dianzani C., Arcaro A., Cetrangolo G.P., Giordano G., Cucci M.A., Graf M., Gentile F. (2018). Lipid Peroxidation-Derived Aldehydes, 4-Hydroxynonenal and Malondialdehyde in Aging-Related Disorders. Antioxidants.

[172] Del Rio D., Stewart A.J., Pellegrini N. (2005). A review of recent studies on malondialdehyde as toxic molecule and biological marker of oxidative stress. Nutr. Metab. Cardiovasc. Dis..

[173] Feng Z., Hu W., Amin S., Tang M.S. (2003). Mutational spectrum and genotoxicity of the major lipid peroxidation product, trans-4-hydroxy-2-nonenal, induced DNA adducts in nucleotide excision repair-proficient and -deficient human cells. Biochemistry.

[174] Hu W., Feng Z., Eveleigh J., Iyer G., Pan J., Amin S., Chung F.L., Tang M.S. (2002). The major lipid peroxidation product, trans-4-hydroxy-2-nonenal, preferentially forms DNA adducts at codon 249 of human p53 gene, a unique mutational hotspot in hepatocellular carcinoma. Carcinogenesis.

[175] Cadenas E., Packer L., Traber M.G. (2016). Antioxidants, oxidants, and redox impacts on cell function-A tribute to Helmut Sies. Arch. Biochem. Biophys..

[176] Sies H. (2020). Oxidative Stress: Concept and Some Practical Aspects. Antioxidants.

[177] Valko M., Rhodes C.J., Moncol J., Izakovic M., Mazur M. (2006). Free radicals, metals and antioxidants in oxidative stress-induced cancer. Chem.-Biol. Interact..

[178] Lichtenberg D., Pinchuk I. (2015). Oxidative stress, the term and the concept. Biochem. Biophys. Res. Commun..

[179] Phaniendra A., Jestadi D.B., Periyasamy L. (2015). Free radicals: Properties, sources, targets, and their implication in various diseases. Indian J. Clin. Biochem..

[180] Han X., Zhuang Y. (2021). PM2.5 induces autophagy-mediated cell apoptosis via PI3K/AKT/mTOR signaling pathway in mice bronchial epithelium cells. Exp. Med..

[181] Liu X., Zhao X., Li X., Lv S., Ma R., Qi Y., Abulikemu A., Duan H., Guo C., Li Y. (2020). PM(2.5) triggered apoptosis in lung epithelial cells through the mitochondrial apoptotic way mediated by a ROS-DRP1-mitochondrial fission axis. J. Hazard. Mater..

[182] Wang Y., Wu T., Tang M. (2020). Ambient particulate matter triggers dysfunction of subcellular structures and endothelial cell apoptosis through disruption of redox equilibrium and calcium homeostasis. J. Hazard. Mater..

[183] Jarmuszkiewicz W., Dominiak K., Galganski L., Galganska H., Kicinska A., Majerczak J., Zoladz J.A. (2020). Lung mitochondria adaptation to endurance training in rats. Free Radic. Biol. Med..

[184] So B., Park J., Jang J., Lim W., Imdad S., Kang C. (2021). Effect of Aerobic Exercise on Oxidative Stress and Inflammatory Response During Particulate Matter Exposure in Mouse Lungs. Front. Physiol..

[185] Mai A.S., Dos Santos A.B., Beber L.C.C., Basso R.D.B., Sulzbacher L.M., Goettems-Fiorin P.B., Frizzo M.N., Rhoden C.R., Ludwig M.S., Heck T.G. (2017). Exercise Training under Exposure to Low Levels of Fine Particulate Matter: Effects on Heart Oxidative Stress and Extra-to-Intracellular HSP70 Ratio. Oxidative Med. Cell. Longev..

[186] Pardo M., Shafer M.M., Rudich A., Schauer J.J., Rudich Y. (2015). Single Exposure to near Roadway Particulate Matter Leads to Confined Inflammatory and Defense Responses: Possible Role of Metals. Environ. Sci. Technol..

[187] Tseng C.Y., Wang J.S., Chao M.W. (2017). Causation by Diesel Exhaust Particles of Endothelial Dysfunctions in Cytotoxicity, Pro-inflammation, Permeability, and Apoptosis Induced by ROS Generation. Cardiovasc. Toxicol..

[188] Yuan Q., Chen Y., Li X., Zhang Z., Chu H. (2019). Ambient fine particulate matter (PM(2.5)) induces oxidative stress and pro-inflammatory response via up-regulating the expression of CYP1A1/1B1 in human bronchial epithelial cells in vitro. Mutat. Res. Genet. Toxicol. Environ. Mutagen..

[189] Li Y., Batibawa J.W., Du Z., Liang S., Duan J., Sun Z. (2021). Acute exposure to PM(2.5) triggers lung inflammatory response and apoptosis in rat. Ecotoxicol. Environ. Saf..

